# Algorithmic Accountability and Public Reason

**DOI:** 10.1007/s13347-017-0263-5

**Published:** 2017-05-24

**Authors:** Reuben Binns

**Affiliations:** Department of Computer Science, University of Oxford, Oxford, UK

**Keywords:** Algorithmic accountability, Public reason, Discrimination

## Abstract

The ever-increasing application of algorithms to decision-making in a range of social contexts has prompted demands for algorithmic accountability. Accountable decision-makers must provide their decision-subjects with justifications for their automated system’s outputs, but what kinds of broader principles should we expect such justifications to appeal to? Drawing from political philosophy, I present an account of algorithmic accountability in terms of the democratic ideal of ‘public reason’. I argue that situating demands for algorithmic accountability within this justificatory framework enables us to better articulate their purpose and assess the adequacy of efforts toward them.

## Introduction

Computer algorithms are increasingly used in decision-making in a range of contexts, from advertising to policing, housing and credit. An entity that needs to make some decision—a *decision-maker*—defers to the output of an automated system, with little or no human input. These decisions affect individuals (*decision-subjects*) by conferring certain benefits or harms upon them. This phenomenon has been termed *algorithmic decision-making*, and there are increasing calls to make algorithmic decision-makers *accountable* for their activities.^1^

While accountability is frequently referred to in this context, it is often undefined and used as an umbrella term for a variety of measures, including transparency, auditing and sanctions of algorithmic decision-makers.^2^ This paper focuses on accountability in the following sense (drawing from (Bovens, Goodin, & Schillemans, 2014)): party A is accountable to party B with respect to its conduct C, if A has an obligation to provide B with some justification for C, and may face some form of sanction if B finds A’s justification to be inadequate. In the context of algorithmic decision-making, an accountable decision-maker must provide its decision-subjects with reasons and explanations for the design and operation of its automated decision-making system. The decision-subject can then judge whether this justification is adequate, and if not, the decision-maker may face some kind of sanction or be forced to retract or revise certain decisions.

For instance, a bank deploying an automated credit scoring system might be held accountable by a customer whose loan application has been automatically denied. Accountability in this scenario might consist of a demand by the customer that the bank provide justification for the decision; a response from the bank with an account of how their system works, and why it is appropriate for the context; and a final step, in which the customer either accepts the justification, or rejects it, in which case the bank might have to revise or reprocess their decision with a human agent, or face some form of sanction.

What happens at this final stage of accountability is often not discussed, but it raises a significant ambiguity at the heart of the notion of algorithmic accountability. What kinds of justifications could the decision-maker legitimately expect the decision-subject to be satisfied with? There are many kinds of justifications that could be made, corresponding to a wide range of beliefs and principles. For instance, the bank might justify their decision by reference to the prior successes of the machine learning techniques they used to train their system; or the scientific rigour involved in the development of their psychometric test used to derive the credit score; or, more fancifully, divine providence. The loan applicant might reject such justifications for various reasons; for instance, on account of their being sceptical about the machine learning technique, the scientific method, or the existence of an interventionist God, respectively. If decision-maker and decision-subject disagree over the adequacy of the justifications provided, how might that conflict be resolved? Might decision-makers sometimes be justified in imposing algorithmic decisions that some decision-subjects find objectionable, or should the latter’s objections always take precedence? How are we to reconcile differing, but legitimate, epistemic and ethical standards to which algorithmic decisions are held?

The notion of algorithmic accountability, whatever its merits, does not resolve these questions; there will always be a plurality of reasonable views on the epistemic and moral status of an algorithmic decision-making system. This paper will argue that this problem is an instance of a more general problem, long-debated in moral and political philosophy. In democratic societies, there is a tension between on the one hand, the need for universal political and moral rules which treat everyone equally; and on the other, the possibility that reasonable people can disagree about the very matters of knowledge, value and morality on which those rules might be decided. An answer to the problem of plural values in algorithmic accountability might therefore be found amongst the responses that political philosophers have offered to this more general problem. In particular, I argue that the notion of *public reason*—roughly, the idea that rules, institutions and decisions need to be justifiable by common principles, rather than hinging on controversial propositions which citizens might reasonably reject—is an answer to the problem of reasonable pluralism in the context of algorithmic decision-making.^3^

Section 2 provides a brief overview of algorithmic decision-making, concomitant problems of opacity, accountability and the ways in which it necessarily embeds epistemic and ethical assumptions which might result in conflict. Section 3 introduces the notion of public reason and outlines its relevance to algorithmic accountability. Section 4 considers potential challenges and limitations, before some concluding remarks.

## The Rise of Algorithmic Decision-Making

This section provides a cursory overview of recent developments in algorithmic decision-making and the ways it is likely to embed epistemic and normative assumptions which are liable to generate the kinds of conflicts between decision-makers and decision-subjects described above.

Society is increasingly driven by intelligent systems and the automatic processing of vast amounts of data. The mediation of life through computation means that predictions, classifications and decisions can be made about people, on the basis of algorithmic models trained on large datasets of historical trends. Personalised platforms build detailed profiles of their user’s attributes and behaviour, which determine the content they view, the products they see and search results they receive (Tufekci, 2014; Sweeney, 2013). Where borrowers were once evaluated for financial loans on a narrow range of historical and qualitative factors, they may now face opaque assessments based on a wide range of seemingly unrelated attributes (Deville, 2013). For instance, online lenders observe behaviours that have been found to correlate with creditworthiness, such as the speed with which potential borrowers scroll through their website, or whether they use capital letters correctly when filling forms (Lobosco, 2013). Employers now use similar systems to choose their employees, monitoring their activity to keep them productive and healthy and predicting their failure, success, resignation, or even suicide, so that early steps can be taken to mitigate the risks (Kim, 2015).

All of these systems are ‘algorithmic’ in the sense that they take in certain inputs and produce certain outputs by computational means. Some of them involve explicitly programmed steps, in which existing knowledge about the world is formally represented, enabling software agents to make inferences and reason on the basis of that knowledge (see e.g. (Shadbolt, Motta, & Rouge, 1993)). Others are based on ‘machine learning’, a more recent paradigm in artificial intelligence (see e.g. Russel & Norvig, 2010 )). Machine learning involves training models with learning algorithms, using large datasets of relevant past phenomena (often generated as a by-product of digitally-mediated human activity), in order to classify or predict future phenomena. While these two approaches differ in how they derive their predictive and classificatory functions, they can both be considered examples of algorithmic decision-making systems in so far as they automatically derive decision-relevant outputs from given inputs.

### Algorithmic Decision-Making Necessarily Embodies Contestable Epistemic and Normative Assumptions

Replacing human decision-makers with automated systems has the potential to reduce human bias (Zarsky, 2016; Sandvig, 2015), but both knowledge-based and machine learning-based forms of algorithmic decision-making also have the potential to embody values and reproduce biases (Nissenbaum, 2001). In the case of knowledge-based systems, the knowledge that is fed into the system, and assumptions that are involved in modelling it, may reflect biases of the system designers and data collection process (Wiener, 1960; Weizenbaum, 1972; Dutton & Kraemer, 1980; Friedman & Nissenbaum, 1996). For algorithmic decision-making systems derived from machine learning, there is another potential source of discrimination. If an algorithm is trained on data that are biased or reflect unjust structural inequalities of gender, race or other sensitive attributes, it may ‘learn’ to discriminate using those attributes (or proxies for them). In this way, decisions based on machine learning algorithms might end up reinforcing underlying social inequalities (Kamiran & Calders, 2012; Bozdag, 2013; Diakopoulos, 2015; Sandvig, Hamilton, Karahalios, & Langbort, 2014; Barocas & Selbst, 2016). This kind of problem might arise when predictive models are used in areas like insurance, loans, housing and policing. If members of certain groups have historically been more likely to default on their loans, or been more likely to be convicted of a crime, then the model may give a higher risk score to individuals from those groups. The emerging fields of ‘discrimination-aware data mining’ (DADM), and ‘fairness, accountability and transparency in machine learning’ (FAT-ML) explore various techniques by which organisations can identify such harms and embed ethical constraints such as fairness into their systems.^4^

The kinds of implicit values inherent in automated decision-making systems are contestable on both epistemic and normative grounds.

First, many epistemic aspects of algorithmic models are open to question in various contexts. These include questions which are internal to the practice of algorithm design and machine learning, such as whether a model is generalizable, over-fitted or over-trained, and other questions relating to the performance of machine learning algorithms (Japkowicz & Shah, 2011). Externally, there may also be more fundamental epistemological questions raised by algorithmic decision-making. For instance, should we consider the outputs of machine learning-based models to be on a par with the kind of models potentially gained from the sciences, or are they akin to useful guesswork (Pietsch, 2016)? Does it matter if they cannot distinguish causation from correlation (Mckinlay, 2017)? Whenever an entity deploys an algorithmic system, they are taking an implicit stance on at least some of these questions. Sometimes these stances are made explicit; for example, Peter Norvig, author of a popular textbook on machine learning, encourages data scientists to remember that ‘essentially, all models are wrong, but some are useful’ ((Halevy, Norvig, & Pereira, 2009) quoting (Box & Draper, 1987)). Similarly, policymakers might argue that the inability of machine learning-based models to provide causal explanations is acceptable in cases where they only require the ability to predict (Kleinberg, Ludwig, Mullainathan, & Obermeyer, 2015). The point here is not to argue for or against any of these particular positions; but rather, to show that algorithmic decision-making systems necessarily refer to contestable epistemological claims which may need justifying and about which people might reasonably disagree.

Second, setting aside these epistemic questions, anyone deploying algorithmic models inevitably also engages a set of normative principles (at least implicitly). Whether or not organisations explicitly attempt to include discrimination-detection and fairness constraints in their models, it is clear the deployment of algorithmic systems inevitably embeds certain ethical assumptions. Whether it is fair to treat an individual in a certain way on the basis of aggregate analysis of the behaviour of other people is a nuanced moral question, likely to depend on multiple contextual factors. And when attempting to modify a model to remove algorithmic discrimination on the basis of race, gender, religion or other protected attributes, the data scientist will inevitably have to embed, in a mathematically explicit way, a set of moral and political constraints.^5^

We can conclude that algorithmic decision-makers (implicitly or explicitly) always embed epistemic and normative assumptions. Since algorithmic accountability (in the sense defined above) involves providing reasons, explanations and justifications for their decisions, one would expect that these assumptions should form a substantial part of the content of a decision-maker’s account.

### Algorithmic Accountability Aims to Draw Out Embodied Values

The need to draw out these assumptions is reflected in recent demands for algorithmic accountability. Politicians, civil society, regulators and academics have called for those who implement such systems to reveal information about how they work. Giving decision-subjects the right to understand the logic behind these systems is seen as a ‘critical right for the profiling era’ (Hildebrandt, 2012), and ‘a first step toward an intelligible society’ (Pasquale, 2011). Reflecting these concerns, many privacy, data protection and freedom of information laws contain various measures to compel organisations to reveal the systems they deploy, what data they collect, the models they infer and how they are used. The 1995 EU Data Protection Directive and the forthcoming General Data Protection Regulation both include a right for individuals to demand an account of the ‘logic’ behind automated decisions made about them (Articles 13.2(f), 14.2(g), and 15.1(h) of the GDPR).^6^ These regulations aim to enable citizens to scrutinise and challenge the otherwise opaque logic of these systems.

If the aim of these measures is to allow accountability, and accountability involves the provision of reasons, explanations and justifications, then this ought to involve drawing out these implicit epistemic and normative standards. To return to the credit scoring example, the bank might articulate the provenance of their data, defend their modelling assumptions, their positive and negative error rates, and the reasonableness of their thresholds for denying or awarding loans. Additionally, they might make reference to any normative standards that are embedded in the design of the algorithm. For instance, they might refer to any anti-discrimination measures incorporated within the model to prevent it from giving higher credit risk scores to members of groups which have historically been discriminated against (using the techniques described above).

### The Dilemma of Reasonable Pluralism

This final stage of accountability raises a significant challenge. While such reasons, explanations and justifications may satisfy some affected individuals, there is no guarantee that the implicit epistemic and normative standards that are appealed to will be acceptable to all. System operators will offer explanations that appeal to standards which they themselves endorse; but which may not be accepted by those whom the decision affects. Divergence between epistemic standards seems entirely legitimate, for instance, given widespread debates about the soundness and robustness of machine learning models (see, for instance, (Szegedy et al., 2013; Nguyen, Yosinski, & Clune, 2015)). Differences of opinion about the normative standards that ought to be embedded in algorithmic decisions seem even more likely, given general differences of moral opinion in any population, and particular differences regarding affected domains like insurance, housing, and policing. There is therefore likely to be a gap between merely providing reasons and explanations for an algorithmic decision-making system’s output, and providing adequate justification for them that will be acceptable to affected decision-subjects. In such cases, there will be an impasse, due to the differing epistemic and normative standards of the system operator and the subject of the decision.

This presents a dilemma about whose standards ought to prevail; those of the algorithmic decision-maker or the decision-subject? Neither is likely to be satisfactory. If it is the former, algorithmic accountability might become a ritualised compliance procedure, used as a rubber stamp to provide superficial legitimacy to the outputs of a system. On the other hand, if we give primacy to the affected individual’s standards, even the most accurate and ethically responsible use of algorithmic decision-making might be objected to by individuals with very different standards. Giving absolute primacy to either the decision-maker or the decision-subject would render algorithmic accountability too one-sided, allowing one party to hold the other to standards that they could not reasonably accept.

Such epistemic and normative differences seem inevitable and likely; and if algorithmic accountability aims to promote legitimacy, then, we need a better account of how to resolve them. In what follows, I propose a reconstructed defence of algorithmic accountability, grounded in the notion of public reason.

## Algorithmic Accountability as Public Reason

I argue that in order to respond to these challenges, proponents of algorithmic accountability should look to the democratic political ideal of *public reason*. In this section, I briefly introduce the notion of public reason, before explaining its relevance to the notion of algorithmic accountability.

### Public Reason: A Brief Overview

Public reason is a concept rooted in the early-modern political philosophy of Rousseau and Kant, and more recently revitalised in the work of Rawls, Habermas and others.^7^ Quong defines it as the requirement that:

‘Our laws and political institutions must be justifiable to each of us by reference to some common point of view, despite our deep differences and disagreements.’ (Quong, 2013).

Public reason attempts to resolve the tension between the need for universal political and moral rules which treat everyone equally, and the idea that reasonable people can disagree about certain matters such as value, knowledge, metaphysics, morality or religion. If the rules that we impose upon each other are only justifiable by appealing to beliefs that some may reasonably disagree with, those who disagree become subject to the political will of others. Public reason therefore proposes that universal rules must be justifiable on grounds that are suitably public and shared by all reasonable people in the society, and without appeal to beliefs that are controversial.

The notion therefore depends on the possibility of a universally reasonably acceptable separation between shared and non-shared matters of belief. Citizens must agree that a certain set of beliefs are universal enough that every reasonable person can be expected to agree with them, while others are too controversial (even if some of them may be true). For instance, suitably universal principles might include equal basic liberty, equality of opportunity and a just distribution of income and wealth. These may be contrasted with the non-universal contents of religious, metaphysical, moral or political doctrines, which cannot form the basis of an overlapping consensus of common principles. This distinction between universal and doctrinal beliefs explains the intuition that “even if the Pope has a pipeline to God’s will, it does not follow that atheists may permissibly be coerced on the basis of justifications drawn from the Catholic doctrine” (to use an example from David Estlund (Estlund, 2008)).

While the demarcation between universal and doctrinal beliefs is recognised by proponents of public reason as a difficult challenge, it is seen as a necessary precondition for a legitimate liberal democracy, and a question various political philosophers have attempted to answer (e.g. (Rawls, 1997)). Typically, the kinds of beliefs that may be appealed to in the course of public reason are normative, but on some accounts, they might also include epistemic matters, such as sufficiently widely accepted scientific knowledge (Rawls, 1996; Jasanoff, 2012).

Public reason has primarily been conceived as a constraint on the rules which justify coercion by the state, usually in the form of legislation. However, on some accounts, it applies not only to political institutions, legislators and judges, but also to private entities and beyond (Quong, 2013). Indeed, for some, it is not just a constraint that applies to ‘political rules’ so construed, but rather a constraint on the legitimate exercise of decision-making power in general (Gaus, 2011).

### Public Reason as a Constraint in Algorithmic Accountability

Equipped with this basic notion of public reason, we can begin to flesh out how it might provide a potential resolution of the pluralist dilemma facing algorithmic accountability. In cases where decision-makers and decision-subjects are at loggerheads, appealing to conflicting normative and epistemic standards, they might look to shared common principles to resolve the conflict.

This suggests that public reason could act as a constraint on algorithmic decision-making power by ensuring that decision-makers must be able to account for their system’s outputs according to epistemic and normative standards which are acceptable to all reasonable people. Just as advocates of public reason in broad political contexts distinguish between universally acceptable values (e.g. equality) and reasonable but contested beliefs (e.g. theism), so we might derive similar common principles which could help resolve conflicts in standards between decision-makers and decision-subjects.

As in most accounts of public reason, the precise content of these common principles is expected to emerge from a process of reflective equilibrium between equal citizens. The particular form of such a reflective equilibrium is the subject of much debate within political philosophy, where there is significant disagreement about its scope, content and basis. Such disagreements are likely to remain in the algorithmic context. Thus, in advocating a public reason-flavoured form of algorithmic accountability, I do not wish to presuppose any particular form of public reason. Nevertheless, it is still possible to explore how a reassertion of the demands of public reason within the process of accountability could prove useful in several ways.

#### Reasserting Universal Principles Against Biases Inherited by Code

First, public reason would be useful in algorithmic versions of the ‘analogue’ conflicts traditionally discussed in political philosophy. Various forms of algorithmic discrimination discussed above are likely to fall into this category. Human biases which violate public reason may be replicated when they are present in the historical data used to train a machine learning algorithm. For instance, some landlords might systematically deny housing to certain tenants on religious grounds. An algorithmic system deployed by a rental agency to score applicants trained on this data may well replicate such biases unintentionally. If those human decisions would not have withstood scrutiny under the criteria of public reason, then their algorithmic descendants likely will not either (unless care has been taken to address and mitigate the biases in training data). Reification in code should not provide such biases with an escape from these constraints.

#### Ensuring Articulation in Accountability

Second, it would help in cases where the legitimacy of a decision is unclear because the decision-maker’s explanation for their algorithmic system is insufficiently developed, poorly articulated or unclear. Where no reference to justificatory principles (whether epistemic or normative) is offered, decision-subjects may lack the means to evaluate the automated decision-making system. By asserting the need to demonstrate compatibility with universally acceptable principles, public reason forces decision-makers to consider the ethical and epistemic aspects of their algorithmic systems ex ante.

#### Navigating the Boundary Between Public and Private Decision-Making

Existing debates about public reason and potential conflicts with private conscience could also prove useful in trying to navigate some delicate boundaries, such as that between discrimination and personalisation. While treating people differently on the basis of a protected characteristic is usually against the universally accepted principles of public reason, there are some situations in which decisions are not subject to those demands. For instance, when choosing a romantic partner, forms of discrimination which would normally be considered illegitimate are generally accepted. Similar exceptions are likely to be made when algorithmic systems are deployed to similar ends; there is no outcry over the fact that algorithms used to match people on internet dating services ‘discriminate’ by gender and sexuality. But other cases may not be so clear-cut. Similarly, there may be important differences in the extent to which public and private organisations are subject to the demands of public reason. Democratically elected governments may rightly be held to stricter standards than private companies, although the latter are still often required to justify their actions by reference to publicly acceptable principles. For these reasons, algorithmic accountability in the public and private sector is likely to differ in nature and scope. But the navigation of these boundaries is already usefully addressed within the theory of public reason.

#### Clarifying the Epistemic Standards of Justification

Public reason may also help to clarify the kinds of epistemic standards required for an adequate justification. For instance, a controversy may arise over whether the predictions an automated system makes are based on causal or correlative relationships between variables. This may be morally relevant if causal relationships are considered to be more legitimate grounds on which to base decisions than mere correlations (Gandy, 2010). To use an example from a recent European Union court decision, being a woman may be correlated with being a more responsible driver, but it is not the ‘real cause’ of responsible driving (which might instead be some neurological or psychosocial dispositions which are more prevalent amongst women) (Schanze, 2013). On this line of thought, an algorithmic decision-making system which operated on the basis of the mere correlation between gender and driving behaviour would be less justifiable than one which operated on a genuinely causal relationship. Conflicts might therefore arise as a result of reasonable disagreements over the nature of the relationships a given algorithmic model is capable of uncovering.^8^ In such cases, consideration of the acceptability of certain scientific claims in the realm of public reason may be helpful. The putative existence of a causal link between two variables may or may not be an example of what Rawls terms a ‘plain truth’, ‘widely accepted, or available, to citizens generally’ and therefore an acceptable ground for differential treatment (Rawls, 1996).

#### Constraining Algorithmic Decision-Subjects as Well as Decision-Makers

Finally, it is worth noting that public reason may not only be a constraint on decision-makers; it might also place constraints on the kinds of grievances a decision-subject can expect to receive sympathy for. Consider a member of a privileged group who had previously benefited from some bias, say, being given preferential access to housing by landlords on account of their religion. Imagine an algorithmic tenant scoring system is created, and those historic biases are scrubbed from the training data in order to prevent their algorithmic replication and ensure the distribution of automated scores is fair. The previously unfairly privileged individual could not complain about their newfound difficulty in securing housing. Membership of a religious group is not a universally acceptable reason for favourable treatment in housing. To have their complaint heard, they would need to be able to ground it in terms of public reason, which would likely fail if the change in relative eligibility were itself justified by public reason.

## Objections, Limitations and Challenges

I now consider two potential limitations and challenges to the idea of public reason as a constraint on algorithmic decision-making.

### Algorithmic Decision-Making Is Already Subject to Public Reason via Substantive Laws

Requiring public reason at the level of algorithmic accountability might seem redundant if it is already present via other forms of regulation. If algorithmic decision-making is already regulated in substantive ways, and those regulations (in a democratic society) are already subject to constraints of public reason, then public reason is already exerting its constraining force. Why attempt to reassert it at a local level?

It is true that public reason will already (ideally) have entered into the legal regulation of algorithmic decision-making through a democratic legislative process. But even so, it may be worth reasserting it at the level of particular systems, embedded in particular contexts, for two reasons.

First, the legislative process is ill-suited to anticipate all the complex and dynamic processes through which an algorithmic decision-making system might evolve. It may not be obvious that a particular law (e.g. non-discrimination) is likely to be violated until such time as the decision-maker is forced to account for their system.

Second, the very act of reasserting the demands of public reason at this level forces the decision-maker to ensure that they have fully articulated the goals of their system and the necessary constraints on the pursuit of those goals. Instructing a system to maximise some outcome without specifying appropriate constraints could lead to a wide range of violations of public reason.^9^ By requiring that the selection of goals and constraints be in line with principles of public reason, potential legal violations may rise to the surface. Accountability serves as an additional layer of enforcement of substantive laws; it is a complement to legal regulation, rather than a replacement for it.

### The Problem of Opacity

A second potential challenge to this model of accountability is raised by the opacity of certain algorithmic decision-making systems. While knowledge-based systems can provide clear explanations of the reasoning behind their outputs (Wick & Slagle, 1989; Gregor & Benbasat, 1999), models trained by machine learning algorithms may not (Lipton, 2015). The lack of interpretability in such algorithmic decision-making systems therefore threatens the ability of decision-makers to account for their systems (Neyland, 2007; Anderson, 2011; O’Reilly & Goldstein, 2013; Burrell, 2016; Ananny & Crawford, 2017 ) . This may lead to ‘algocracy’, in which ‘the legitimacy of public decision-making processes’ is thwarted by ‘the opacity of certain algocratic governance systems’ (Danaher, 2016).

While the lack of interpretability of certain models is indeed a challenge, the prospect for intelligible explanations in general is not hopeless. First, many algorithmic decision-making systems do not rely on inscrutable deep learning and neural networks, but less complex and more interpretable models such as decision trees. Considering the wide range of machine learning methods available, there are trade-offs to be made between interpretability and accuracy (Bratko, 1997; Ribeiro, Singh, & Guestrin, 2016). But even in cases of complex multi-layered models, the problem of opacity may be overestimated; there are various promising approaches to explaining the specific outputs of a model without attempting to open its ‘black box’ (Ribeiro et al., 2016; Datta, Sen, & Zick, 2016).

Even if models do prove to be unavoidably opaque, public reason may also be the vehicle through which we resolve whether this opacity is in fact a problem. In cases where decision-makers can provide no other explanation for a decision than that, say, a neural net produced it, we may decide that their justification fails by default. As Ananny and Crawford argue, ‘if a system is so complex that even those with total views into it are unable to describe its failures and successes, then accountability models might focus on the whether the system … should be built at all’ (Ananny & Crawford, 2017 ). Furthermore, accounting for a system is about more than simply explaining its outputs. In some cases, what matters will not be how a system arrived at a certain output, but what goals it is supposed to serve. Knowing whether a search engine is optimised for popularity, authenticity or novelty of results may be more important than knowing exactly how it achieves those goals. In such cases, the opacity of the model may be less of a barrier to accountability.

## Conclusion

Calls for algorithmic accountability have grown over recent years. They are reflected in the ongoing efforts of policymakers and in data protection law (e.g. Articles 13-15, 20 of the GDPR). The obligation for entities who deploy algorithmic systems to be open about how they operate, and to be held accountable for their algorithmically driven decision-making is an important safeguard against abuse.

But less has been said about what should happen after algorithmic decisions are made accountable. When an organisation is forced to account for their systems, what are we to make of their accounts? What kinds of accounts should we accept as valid? Public reason offers a partial answer.

Just as democratic citizens have the right to scrutinise and hold account the exercise of political power, so algorithmic constituents have the right to scrutinise and hold account the exercise of algorithmic power. But when conflicts arise, between the entities making and implementing algorithmic decisions, and those who are subject to them, how should these conflicts be resolved? On the one hand, we cannot expect citizens to abide by the outputs of algorithms whose epistemic and normative standards they may reasonably not endorse. And yet on the other, we must also offer some positive criteria by which an entity could possibly succeed in offering a satisfactory account of their algorithmic system. The answer suggested by public reason is that the entity wishing to implement its algorithm must be able to account for its system in normative and epistemic terms which all reasonable individuals in society could accept.

## Footnotes

1 See e.g. (Diakopoulos, 2016), and section 2 for further examples.

2 Ibid.

3 See e.g. (Quong, 2013) for an overview.

4 See e.g. (Pedreschi, Ruggieri, & Turini, 2009 ).

5 See e.g. (Joseph, Kearns, Morgenstern, Neel, & Roth, 2016).

6 For discussion of the details of the so-called ‘right to explanation’, see (Goodman & Flaxman, 2016) and, contra, (Wachter, Mittelstadt, & Floridi, 2016).

7 See e.g. (Rawls, 1997; Raz, 1998; Hawbermas, 1993).

8 For discussion of how machine learning might uncover causal relationships, see e.g. (Pearl, 2009). See (Pietsch, 2016) for its implications for the scientific method and (Mckinlay, 2017) on causal explanations.

9 Although, significant challenges in specifying constraints remain; see e.g. (Bostrom, 2003).
